# Data on assessment of *Acinetobacter* sp. on growth and yield parameters of rice under field condition

**DOI:** 10.1016/j.dib.2017.02.039

**Published:** 2017-02-21

**Authors:** G. Muralidharan, A. Gandhi

**Affiliations:** a Department of Botany, Annamalai University, Annamalai Nagar 608002, Tamilnadu, India; b H.H. The Rajah׳s College, Pudukkottai 622001, Tamilnadu, India

**Keywords:** Rice, Field, Zinc solubilizing bacteria, *Acinetobacter* sp

## Abstract

In this paper, we presented the information on growth and yield parameters of rice genotype (BPT5204 and IR64) in field condition. Investigated strains are related to the research article “Assessment of zinc solubilizing potentiality of *Acinetobacter* sp. isolated from rice rhizosphere” [1]. The article describes the inoculation effect of *Acinetobacter* sp. on rice genotypes. The statistical analysis of the experimental data was carried out using social science SPSS software (ver. 16.0 for windows).

**Specifications Table**

**Table:** 

Subject area	Agronomy, Biology
More specific subject area	Microbiology
Type of data	Table
How data was acquired	Growth and Yield parameters of rice genotype BPT5204 and IR64
Data format	Raw and recorded
Experimental features	5 replicates were used as CRBD design
Data source location	Cuddalore, Tamilnadu, India
Data accessibility	Data available within this article

**Value of the data**

• Data presented here increased the growth and yield parameters of rice varieties in individual as well as combined inoculation of *Acinetobacter* sp. in field condition.
• The data shown here may be used by researchers to understand the performance of bacteria in field condition.
• Data investigated here are useful to the researchers working in the area of plant-microbe interaction, Microbiology and Rhizosphere ecology.

## 1. Data

Two strains of *Acinetobacter* sp. isolated from rice rhizosphere, AGM3 (*Acinetobacter* sp; NCBI Accession Number-KP888315), AGM9 (*Acinetobacter* sp; NCBI Accession Number – KP888316), reported earlier by us as potential zinc solubilizer [1]. The dataset of this article contains three tables (Table 1, Table 2, Table 3) presenting the information on growth and yield parameter of rice genotypes.

## 2. Experimental design, materials and methods

The experiment was conducted at a rice field with long and short duration variety of BPT5204 (150 days) and IR64 (90–120 days), which normally yields 5.5 to 6.5 t/ha^-1^ and 4.0 to 4.5 t/ha^-1^ in 2013–2014 at rice field of Alapakkam: Latitude: 11.3600°N and Longitude: 79.4300°E and Vadalur: Latitude: 11.5573°N and Longitude: 79.5547°E, cuddalore, India. The experiment was performed in a completely randomized block design (CRBD) with five replicates and sub plot sizes of 25 cmx25 cm. The two strains of *Acinetobacter* sp. AGM3 and AGM9 were grown on a nutrient broth for one week. The 25 days old seedlings were uprooted from the nursery and the roots were dipped in the nutrient broth (10^-8^ CFU ml^-1^) for one hour and transplant a row to row and a plant to plant spacing of 25 cm. The *Acinetobacter* (1500 ml; 10^-8^ CFU ml^-1^) were applied once in 20 days until the flowering stage along with the irrigation. Control contained no bacterial strain (only soil). The crop was harvested manually and the growth parameters were observed with five plants from each plot were selected at random from middle of the rows for sampling and labeled. The growth parameters of rice varieties BPT5204 and IR64 such as Plant height, Leaf length, Number of tillers per hill, plant dry matter production and 50 percent flowering. Grain maturity was taken from the labeled plants. The number of days taken from sowing to maturity stage was recorded and presented as days to maturity. The following yield components were recorded at harvest from the randomly selected plants in each field plot are Number of panicle (Productive tillers) per plant, Panicle length, Total number of grain panicle^-1^, Number of filled grains panicle^-1^, 1000 grains weight, Grain yield and Straw yield. (Table 1, Table 2).

## Figures and Tables

**Figure:**
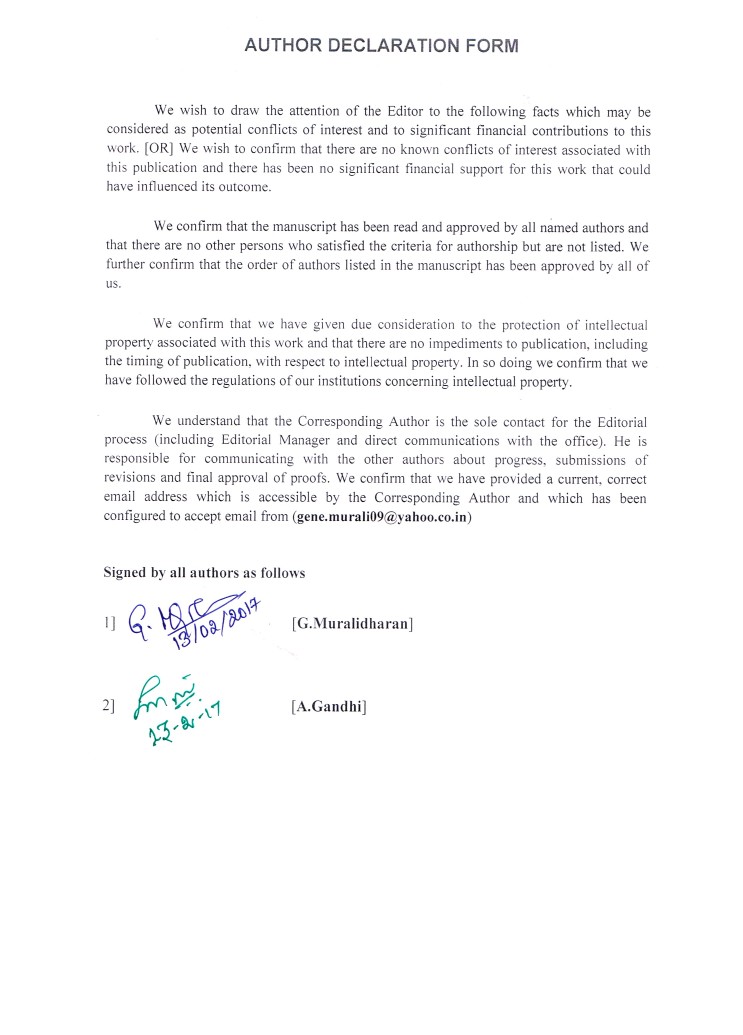


**Table 1:** Growth parameters of rice genotype BPT5204 and IR64 at harvest.

**Treatments**	**Plant height (cm)**		**Leaf length (cm)**		**Number of tillers hill**^**-1**^		**Plant dry matter**		**50% flowering**		**Number of days to maturity**	
	**BPT5204**	**IR64**	**BPT5204**	**IR64**	**BPT5204**	**IR64**	**BPT5204**	**IR64**	**BPT5204**	**IR64**	**BPT5204**	**IR64**
Control	72.12 ±0.35^a^	60.24 ±0.21^a^	64.21 ±0.55^a^	50.76 ±0.15^a^	28.02 ±0.29^a^	31.14 ±0.57^a^	25.61 ±0.40^a^	16.32 ±0.01^a^	101.72 ±0.36^b^	98.82 ±0.25^b^	115.23 ±0.66^c^	89.12 ±0.06^b^
AGM3	74.13 ±0.54^b^ (6.40)	61.53 ±0.4^8a^ (4.10)	65.92 ±0.75^b^ (5.44)	52.22 ±0.24^b^ (4.65)	30.30 ±0.23^bc^ (7.25)	32.58 ±0.50^ab^ (4.58)	27.13 ±0.70^b^ (4.84)	18.23 ±0.04^b^ (6.08)	99.46 ±0.42^a^ (-7.19)	97.02 ±0.01^a^ ( -5.73)	111.21 ±0.19^b^ (-12.79)	86.55 ±0.36^a^ (-8.18)
AGM9	73.56 ±0.62^b^ (4.58)	61.28 ±0.87^a^ (3.31)	65.33 ±0.36^ab^ (3.56)	52.00 ±0.61^b^ (3.95)	29.52 ±0.55^b^ (4.77)	32.69 ±1.17^b^ (4.93)	27.03 ±0.53^b^ (4.52)	18.04 ±0.29^b^ (5.47)	99.80 ±0.18^a^ (-6.11)	97.35 ±0.37^a^ ( -4.68)	112.02 ±0.47^b^ (-10.21)	86.65 ±0.41^a^ (-7.86)
AGM3 +AGM9	74.20 ±0.92^b^ (6.62)	61.86 ±1.59^a^ (5.15)	66.13 ±0.94^b^ (6.11)	53.62 ±0.88^c^ (9.10)	30.69 ±0.85^c^ (8.50)	34.04 ±0.64^b^ (9.23)	27.57 ±0.52^b^ (6.24)	18.36 ±0.32^b^ (6.49)	99.12 ±0.36^a^ (-8.27)	96.90 ±0.42^a^ ( -6.11)	110.46 ±0.86^a^ (-15.18)	86.07 ±0.53^a^ (-6.11)

Data are represented as mean±standard error (n=5), Mean values in each column with the same superscript(s) do not differ significantly by Duncan post hoc multiple comparison tests (P≤0.05). Values within parenthesis represent percent increase (+) or decrease (-) with respective control.

**Table 2:** Yield parameters of rice BPT5204 and IR64 at harvest.

**Treatments**	**Number of panicles (Plant**^**-1**^**)**		**Panicle length (cm)**		**Number of grains panicle**^**-1**^		**Number of filled grains panicle**^**-1**^	
	**BPT5204**	**IR64**	**BPT5204**	**IR64**	**BPT5204**	**IR64**	**BPT5204**	**IR64**
Control	17.16 ±0.11^a^	16.62 ±0.39^a^	18.49 ±0.19^a^	16.13 ±0.36^a^	115.41 ±0.20^a^	111.53 ±0.12^a^	99.52 ±0.34^a^	96.18 ±0.37^a^
AGM3	20.10 ±0.22^b^ ( 9.35)	18.70 ±0.36^bc^ ( 6.62)	19.96 ±1.05^b^ ( 4.68)	17.46 ±0.53^ab^ (4.23)	117.68 ±0.54^b^ (7.22)	113.54 ±0.80^b^ (6.40)	102.69 ±0.56^b^ (9.77)	98.65 ±0.56^b^ (7.86)
AGM9	19.80 ±0.34^b^ (8.4)	18.31 ±0.53^b^ (5.38)	19.68 ±0.80^ab^ ( 3.79)	17.02 ±0.80^b^ (2.83)	117.54 ±0.03^b^ (6.78)	112.83 ±0.82^b^ (4.14)	102.27 ±0.79^b^ (8.43)	98.06 ±0.70^b^ (5.98)
AGM3 +AGM9	20.40 ±0.81^b^ ( 10.31)	19.30 ±0.24^c^ ( 8.53)	20.57 ±0.53^b^ (6.62)	18.00 ±0.44^b^ (5.95)	118.59 ±0.50^c^ (10.12)	113.92 ±0.63^b^ (7.60)	103.01 ±0.71^b^ (10.79)	99.58 ±1.20^b^ (10.82)

Data are represented as mean± standard error (n=5), Mean values in each column with the same superscript(s) do not differ significantly by Duncan post hoc multiple comparison tests (P≤0.05). Values within parenthesis represent percent increase (+) or decrease (-) with respective control.

**Table 3:** Effect of inoculation on 1000 grain weight, straw and grain yield at harvest.

**Treatments**	**1000 grain weight (g)**		**Straw yield (g)**		**Grain yield (g)**	
	**BPT5204**	**IR64**	**BPT5204**	**IR64**	**BPT5204**	**IR64**
Control	15.47 ±0.21^a^	18.47 ±0.37^a^	25.12 ±0.13^a^	21.72 ±0.48^a^	15.75 ±0.39^a^	13.29 ±0.46^a^
AGM3	17.89 ±0.62^b^	20.22 ±0.51^b^	27.73 ±0.23^bc^	23.00 ±0.63^ab^	18.03 ±0.66^bc^	15.84 ±0.42^b^
	(7.70)	(5.57)	(8.30)	(4.07)	(7.25)	(8.11)
AGM9	17.52 ±0.48^b^	19.89 ±0.59^b^	27.15 ±0.68^b^	22.88 ±1.63^ab^	17.56 ±0.31^b^	15.47 ±0.32^b^
	(6.52)	(4.52)	(6.46)	(3.69)	(5.76)	(6.94)
AGM3 +AGM9	18.16 ±0.53^b^	20.51 ±0.46^b^	28.13 ±0.26^c^	23.69 ±0.68^b^	18.59 ±0.41^c^	16.18 ±0.14^b^
	(8.56)	(6.49)	(9.58)	(6.27)	(9.04)	(9.20)

Data are represented as mean±standard error (n=5), Mean values in each column with the same superscript(s) do not differ significantly by Duncan post hoc multiple comparison tests (P≤0.05). Values within parenthesis represent percent increase (+) or decrease (-) with respective control.
